# Syncope as the First Clue to a Congenital Heart Defect

**DOI:** 10.1016/j.acepjo.2025.100208

**Published:** 2025-06-27

**Authors:** Ravi Soni, Victoria Morris, Benjamin Karfunkle

**Affiliations:** Department of Emergency Medicine, University of Texas Health Science Center at Houston, Houston, Texas, USA

**Keywords:** point of care ultrasound, congenital heart disease, heart murmur, syncope

## 1 Case Presentation

A 27-year-old woman with no known medical history presented to the emergency department with complaints of intermittent episodes of dizziness and 2 syncopal events over the past week, both of which occurred during arguments with her spouse. She denied associated dyspnea, chest pain, or lightheadedness prior to the episodes. Physical examination was notable for a midsystolic murmur, with no other abnormal findings.

The vital signs were as follows:

Blood pressure: 121/77

Temperature: 36.6°C

Heart rate: 98 bpm

Respiratory rate: 18 breaths/min

SpO2: 98% on room air

Laboratory tests conducted: Complete blood count, Basic metabolic panel, Troponin, urinalysis, and pregnancy test.

Electrocardiogram with normal sinus rhythm, normal intervals, incomplete right bundle branch block

**Figure 1:**
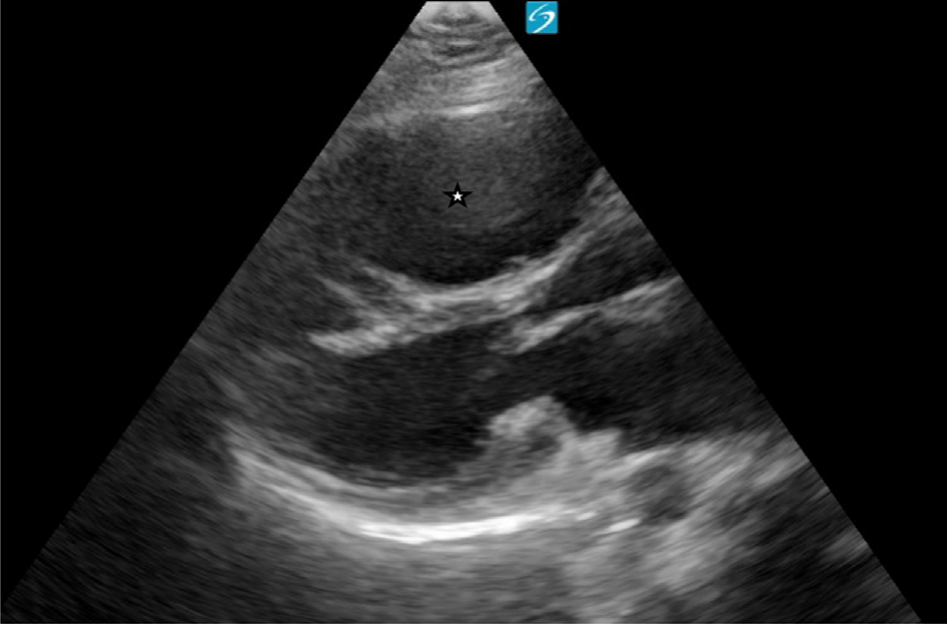
Point-of-care ultrasound (parasternal long-axis view) demonstrating a dilated right ventricle. Image taken by the author.

**Figure 2:**
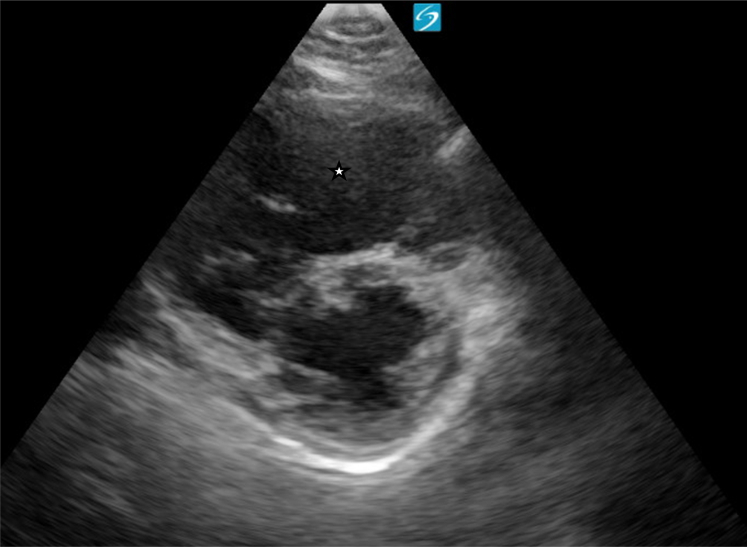
Point-of-care ultrasound (parasternal short-axis view) showing right ventricular dilation. Image taken by the author.

**Figure 3:**
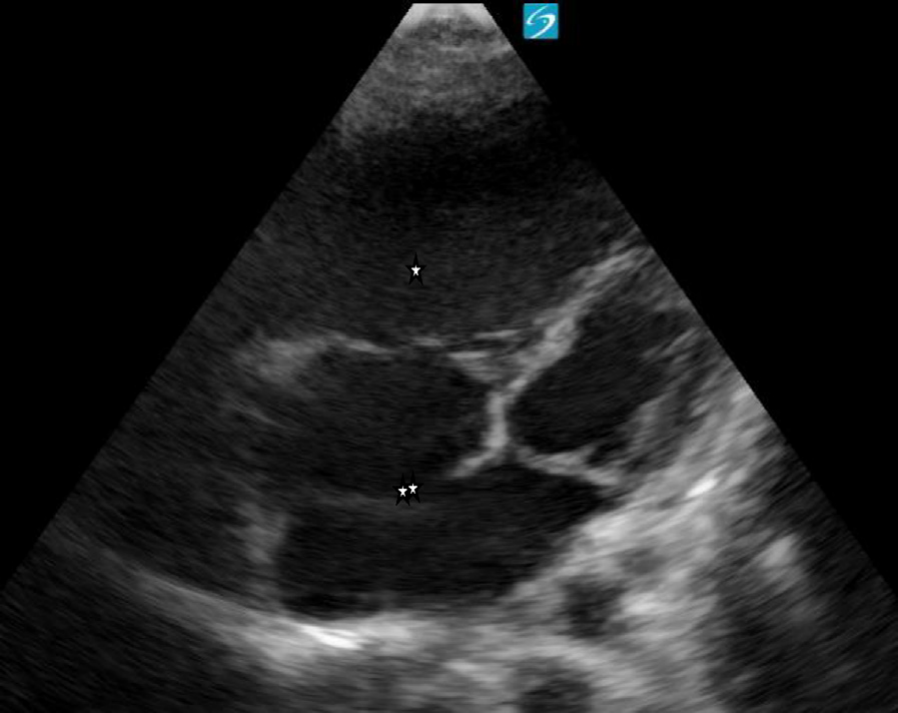
Apical four-chamber view on point-of-care ultrasound revealing dilation of the right atrium and right ventricle (∗), as well as an atrial septal defect (∗∗). Image taken by the author.

**Figure 4:**
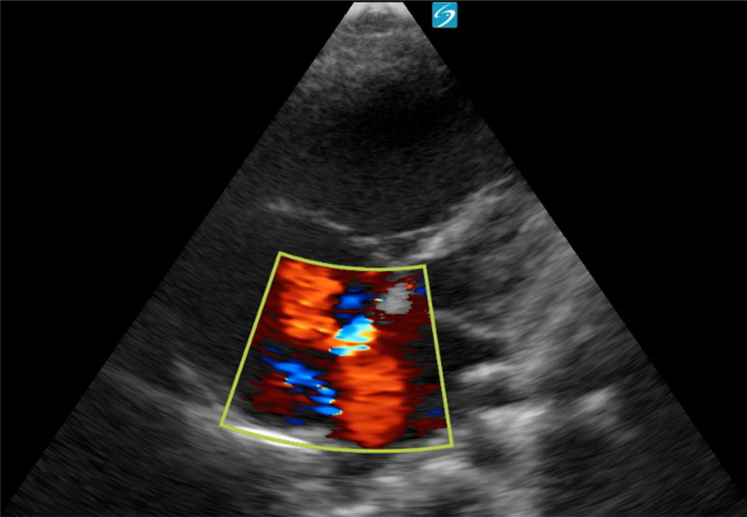
Apical four-chamber view with color Doppler demonstrating left-to-right shunting through an atrial septal defect. Image taken by the author.

**Figure 5:**
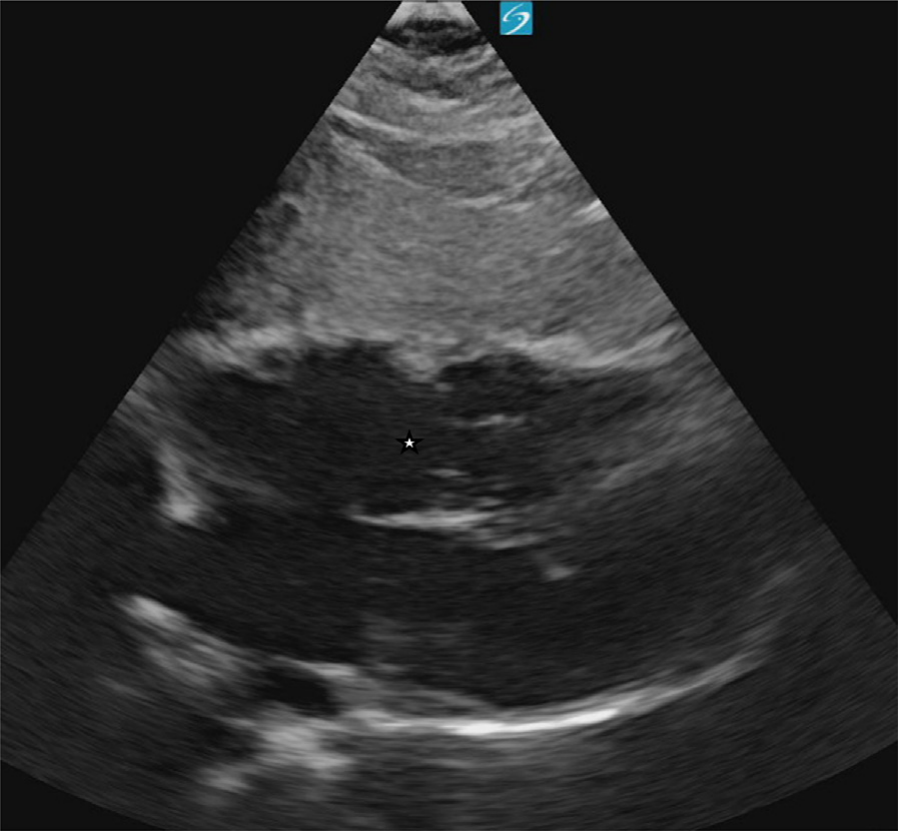
Subxiphoid view showing right heart dilation and evidence of an atrial septal defect. Image taken by the author.

**Figure 6:**
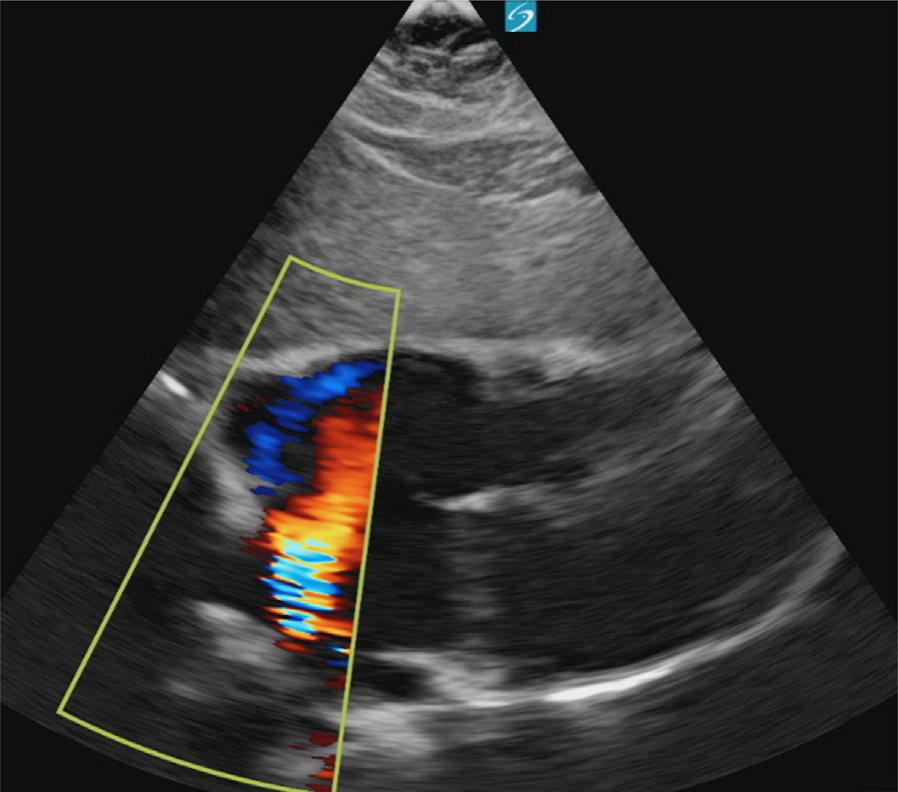
Subxiphoid view with color Doppler revealing right heart dilation and flow across the atrial septal defect. Image taken by the author.

A point-of-care cardiac ultrasound showed the congenital heart defect (Figure 1, Figure 2, Figure 3, Figure 4, Figure 5, Figure 6).

## 2 Diagnosis: Atrial Septal Defect With Evidence of Right Ventricular Overload

An atrial septal defect (ASD) is one of the most common types of congenital heart defects, characterized by persistent communication between the right and left atria after birth,^1^ The majority of cases are diagnosed in early childhood, in utero, or during infancy; however, some may remain undetected until adulthood. Symptom presentation depends on the size of the defect. Although many patients remain asymptomatic, larger defects can result in fatigue, exercise intolerance, dyspnea, palpitations, and signs of pulmonary hypertension.^2^

In this case, the patient’s bedside echocardiogram was concerning for a large ASD with evidence of right ventricular dilation. A formal echocardiogram confirmed the diagnosis, revealing a 3.4 cm ASD with signs of right ventricular volume and pressure overload and a preserved ejection fraction of 60% to 65%. Her labs did not show any acute abnormalities, and her electrocardiogram showed normal sinus rhythm, normal intervals, and an incomplete right bundle branch block. She was transferred for evaluation and intervention by cardiothoracic surgery.

## Funding and Support

By *JACEP Open* policy, all authors are required to disclose any and all commercial, financial, and other relationships in any way related to the subject of this article as per ICMJE conflict of interest guidelines (see www.icmje.org). The authors have stated that no such relationships exist.

## Conflict of Interest

All authors have affirmed they have no conflicts of interest to declare.
